# Using connectome-based models of working memory to predict emotion regulation in older adults

**DOI:** 10.1093/scan/nsad036

**Published:** 2023-07-08

**Authors:** Megan E Fisher, James Teng, Oyetunde Gbadeyan, Ruchika S Prakash

**Affiliations:** Department of Psychology, The Ohio State University, Columbus, OH 43210, USA; Center for Cognitive and Behavioral Brain Imaging, The Ohio State University, Columbus, OH 43210, USA; Department of Psychology, The Ohio State University, Columbus, OH 43210, USA; Center for Cognitive and Behavioral Brain Imaging, The Ohio State University, Columbus, OH 43210, USA; Center for Cognitive and Behavioral Brain Imaging, The Ohio State University, Columbus, OH 43210, USA; National Centre for Healthy Ageing, Peninsula Clinical School and Monash University, Melbourne 3800, Australia; Department of Psychology, The Ohio State University, Columbus, OH 43210, USA; Center for Cognitive and Behavioral Brain Imaging, The Ohio State University, Columbus, OH 43210, USA

**Keywords:** connectome-based predictive modeling, working memory, emotion regulation, aging, fMRI

## Abstract

Older adulthood is characterized by enhanced emotional well-being potentially resulting from greater reliance on adaptive emotion regulation strategies. However, not all older adults demonstrate an increase in emotional well-being and instead rely on maladaptive emotion regulation strategies. An important moderator of age-related shifts in strategy preferences is working memory (WM) and its underlying neural circuitry. As such, individual differences in the neural integrity underlying WM may predict older adults’ emotion regulation strategy preferences. Our study used whole-brain WM networks—derived from young adults using connectome-based predictive modeling—to predict WM performance and acceptance strategy use in healthy older adults. Older adults (*N *= 110) completed baseline assessments as part of a randomized controlled trial examining the impact of mind-body interventions on healthy aging. Our results revealed that the WM networks predicted WM accuracy but not acceptance use or difficulties in emotion regulation in older adults. Individual differences in WM performance, but not WM networks, moderated relationships between image intensity and acceptance use. These findings highlight that robust neural markers of WM generalize to an independent sample of healthy older adults but may not generalize beyond cognitive domains to predict emotion-based behaviors.

## Introduction

Older adulthood is associated with greater reliance on adaptive emotion regulation strategies, such as acceptance and positive reappraisal (Nowlan *et al.*, 2015; Schirda *et al.*, 2016). However, not all older adults demonstrate this overall positive trajectory with individual differences in cognitive abilities, such as attention and working memory (WM), playing a pivotal role in an individual’s preference for and use of select emotion regulation strategies (McRae and Gross, 2020; Pruessner *et al.*, 2020). The Selection, Optimization, and Compensation with Emotion Regulation theory (Urry and Gross, 2010; Opitz *et al.*, 2012) and cognitive control hypotheses (Nashiro *et al.*, 2012; Powers and LaBar, 2019) suggest that age-related declines in prefrontal cortex engagement reduce cognitive control abilities resulting in shifts in emotion regulation processes. Thus, older adults with reduced cognitive abilities may compensate for losses by using less cognitively demanding strategies, like distraction (Scheibe *et al.*, 2015; Martins *et al.*, 2018). Furthermore, cognitive reappraisal—shown to promote emotional health through the reinterpretation of the emotional situation or goals (Aldao *et al.*, 2010)—is the most well-studied strategy to date (Buhle *et al.*, 2014; McRae, 2016). Meta-analytic neuroimaging evidence provides robust support that reappraisal-based strategies recruit brain regions associated with cognitive control (Buhle *et al.*, 2014; Morawetz *et al.*, 2017, 2020), specifically the dorsolateral prefrontal cortices (among other regions; Yaple *et al.*, 2019; Luo *et al.*, 2020).

Although many cognitive functions are implicated in adaptive emotion regulation, some of the strongest empirical evidence supports the critical role of WM (Opitz *et al.*, 2012; Lee *et al.*, 2013; Hendricks and Buchanan, 2016). WM is the process through which the brain stores, prioritizes and actively manipulates information in real time to meet task-relevant goals (Baddeley and Hitch, 1974). Among healthy adults, greater WM is associated with reductions in negative affect during the use of reappraisal and suppression strategies (Hendricks and Buchanan, 2016; Gan *et al.*, 2017). Furthermore, young adults who completed 20 days of WM training exhibited greater reductions in late positive potential amplitudes—an event-related brain potential indexing emotional reactivity—when viewing unpleasant images, which was more pronounced during reappraisal relative to distraction use (Xiu *et al.*, 2018). Not only do these findings highlight that WM is important for adaptive emotion regulation but also suggest that strategies differentially recruit WM. Critically, because WM abilities decline with increasing age (Jenkins *et al.*, 1999; Brockmole and Logie, 2013; Pliatsikas *et al.*, 2019), individual differences in WM may partially account for the divergent patterns of adaptive *vs* maladaptive strategy use in older adulthood.

Considering these findings, the primary goal of this study was to examine whether brain-based markers of WM performance were associated with the prioritization of an adaptive strategy (acceptance) compared with a maladaptive strategy (suppression) during a behavioral task of emotion regulation. Despite limited research on the association between cognitive control metrics and the use of acceptance-based strategies, our prior work has evinced support for WM as an important moderator of mindfulness-based gains on attentional control and emotion dysregulation (Whitmoyer *et al.*, 2020; Samimy *et al.*, 2022). Mindfulness-based interventions, involving the cultivation of sustained attention in a framework that promotes non-judgment and acceptance, is a third-wave psychotherapeutic intervention that prioritizes acceptance of internal phenomenological experience (Shapiro *et al.*, 2006; Chiesa *et al.*, 2014; Lindsay and Creswell, 2017). Our prior studies indirectly underscore the importance of WM as a moderator of improvements in attentional control and reductions in emotion dysregulation resulting from an acceptance-based intervention. Extending our prior work, this study will directly examine whether whole-brain functional connectivity markers of WM are associated with the selective prioritization of using an acceptance-based strategy during an emotionally evocative task.

Given that complex psychological processes, such as emotion regulation, are supported by multiple brain regions, functional magnetic resonance imaging (fMRI) researchers are shifting away from a focus on select cortico-subcortical regions and toward harnessing the variance distributed across the connectome to identify brain-based signatures of such emergent constructs. Connectome-based predictive modeling (CPM; Shen *et al.*, 2017) is one such technique that uses whole-brain functional connectivity and supervised machine learning to identify functional connections between brain regions (i.e. edges) that predict the behavior of interest. Recently, Avery *et al.* (2020) identified a set of 1,674 edges that predicted better WM (high-WM network) and a set of 1,203 edges that predicted poorer WM abilities (low-WM network) in a large sample (*N *= 502) of young adults. These WM CPM (wmCPM) networks predicted individual differences in visual and verbal memory performance composite scores in an independent sample of older adults with amnestic mild cognitive impairment, Alzheimer’s disease, and healthy controls (Avery *et al.*, 2020). Although the edges within the high- and low-WM networks were widely distributed across cortical and subcortical regions, edges within the frontoparietal, subcortical–cerebellar and motor networks showed the strongest contribution to predicting performance (Avery *et al.*, 2020). Interestingly, when the functional anatomy of rest- *vs* task-trained wmCPMs was compared, the task-trained models showed greater involvement of Default Mode Network (DMN) and DMN-associated regions in the high-WM networks compared to the low-WM networks. These findings run counter to a robust literature showing anticorrelations between the DMN and task-positive networks (Fox *et al.*, 2005), although some evidence suggests that task-persistent DMN activity may also be important for WM performance and adjusting to changes in cognitive contexts (Hampson *et al.*, 2006, 2010; Crittenden *et al.*, 2015).

Importantly, because aging is associated with declines in WM (Jenkins *et al.*, 1999; Brockmole and Logie, 2013; Pliatsikas *et al.*, 2019) and changes in the functional architecture of the brain (Rypma and D’Esposito, 2000; Yaple *et al.*, 2019), the edges most relevant for WM may differ for older adults than those identified in the wmCPM networks. Although these networks have successfully predicted WM performance in large independent samples of children and adults (Kardan *et al.*, 2022) as well as two independent samples of people with multiple sclerosis (Manglani *et al.*, 2021), the generalizability of these networks to older adults remains to be seen. Together, these findings suggest the wmCPMs are robust neuromarkers of WM and successfully generalize to other cognitive domains in previously unseen adults. Yet, the generalizability of these WM networks for healthy older adults has not been established.

As such, the current study will use the wmCPM networks derived from Avery *et al.* (2020) to examine whether they predict WM performance and acceptance strategy use in our independent sample of healthy older adults. For our primary aim, we hypothesized that greater network strength in the combined-WM network would be associated with better WM performance and greater use of an acceptance-based strategy. Additionally, in an exploratory aim, we examined whether wmCPM network strength moderated the relationship between image intensity and acceptance strategy use. Specifically, we hypothesized that individuals with greater combined-WM network strength would exhibit greater use of acceptance during high-intensity image trials. All hypotheses and statistical analyses were pre-registered on the Open Science Framework (https://osf.io/vdb24) prior to conducting the following analyses.

## Methods

The data used for the current study were collected as part of a randomized controlled trial designed to examine the effects of two mind-body interventions on attentional control in healthy older adults (Prakash *et al.*, 2022). An a priori power analysis was conducted using the RStudio ‘pwr’ package (Champely, 2020) to assess the sample size needed to test the primary aims. Our power analysis was based on the observed effect size (*r *= 0.37) between the *N*-back 2-back accuracy predicted and observed scores reported in Avery *et al.* (2020). Using an effect size (*r*) of 0.37 and an alpha level of 0.05, a total of 55 participants would be needed to yield an estimated power of 0.80.

### Participants

Participants were initially recruited from the greater Columbus, Ohio area through a combination of advertisements and community outreach events. Detailed inclusionary and exclusionary criteria for the parent study have been published in the study protocol paper (Prakash *et al.*, 2022). Briefly, participants were between the ages of 65 and 85 years, were right-handed and were required to have a corrected visual acuity of 20/40 or better, adequate hearing and self-reported fluency in spoken English. Participants who had previously engaged in formal mindfulness training or currently had a formal meditation practice were ineligible for the study. Participants were excluded if they exhibited cognitive impairment levels consistent with dementia and had been diagnosed with a terminal illness, a neurological or inflammatory disorder, a psychotic or substance abuse disorder or a learning disability. Additionally, participants diagnosed with a psychiatric disorder in the last 2 years by a mental health professional and those scoring $ \ge $20 on the Center for Epidemiological Studies Depression-Scale (CES-D; Radloff, 1977) were excluded from the study. Participants currently taking medications belonging to any of the following drug classes were also excluded: sedatives, selective serotonin reuptake inhibitors, benzodiazepines, barbiturates, sedative-hypnotics, anti-inflammatories, chemotherapies and any drugs altering brain function or enhancing cognitive performance. The study procedures were approved by The Ohio State University Institutional Review Board (IRB no.: 2017H0223). A total of 173 participants signed informed consent, of which 23 were excluded for not meeting the inclusion criteria, declining to participate, and for other reasons. Of the 150 participants who completed the baseline behavioral assessments, 40 did not complete the neuroimaging portion of the study because of MR contraindications or other reasons. Thus, 110 older adults who completed both the baseline behavioral and neuroimaging assessments were included in the current study.

### Procedures

Participants were enrolled in cohorts of 15–32 people for the parent study between August 2018 and May 2022. Prior to the start of the intervention sessions, participants completed a series of assessments to establish a baseline profile of cognitive, affective and neural functioning. First, participants attended a neuropsychological assessment lasting ∼3 h in which they completed a series of cognitive and emotional tasks. Next, participants were emailed a link to a battery of self-report questionnaires which they completed via Qualtrics (Qualtrics, 2019). Finally, participants attended a neuroimaging assessment session during which they underwent MRI safety screening and training in the cognitive tasks. Then the participants completed a series of structural and functional MRI scans lasting 90 min. Participants were paid $10 per hour for the behavioral assessments, $15 per hour for neuroimaging assessments and an additional $10 for completing the questionnaires.

### Measures

#### Emotion regulation choice task

Participants completed an adapted version of the emotion regulation choice (ERC) task (Sheppes *et al.*, 2011) during the behavioral assessment. The ERC task required participants to select between two emotion regulation strategies (acceptance or suppression) to regulate their emotions to 12 high-intensity and 12 low-intensity unpleasant images from the International Affective Picture System (Lang *et al.*, 1999). Prior to completing the task, participants read about each strategy followed by six practice trials (three trials on acceptance and three trials on suppression). During these practice trials, participants were instructed to verbally describe how they were using each emotion regulation strategy in response to the practice images. If a participant’s description reflected a different emotion regulation strategy (e.g. cognitive reappraisal and distraction), experimenters provided additional clarification and asked participants to describe how to use the strategy using the same practice image. Once participants demonstrated an adequate understanding of the two strategies, they completed 24 task trials. During each trial, participants were presented with a preview of the image (1500 ms), followed by a slide asking them to select either acceptance or suppression (infinite ms), followed by the same image again for a longer duration (12 000 ms) in which they were instructed to regulate their emotions to the image using the strategy they selected.

Acceptance was defined as letting one’s feelings come and go without trying to control or avoid them, whereas suppression was defined as pushing one’s feelings down or out of their mind. Following each image trial, participants were asked three questions about their emotional experience. Overall acceptance use was calculated as the percentage of total trials (24 trials) in which the participant selected acceptance to regulate their negative emotions. Overall acceptance use scores ranged between 0% and 100%, such that higher scores indicated a greater preference for acceptance use. Similarly, acceptance use on low- and high-intensity images was calculated as the percentage of low- and high-intensity images (12 trials each) in which the participant selected acceptance. Acceptance use scores for low- and high-intensity images ranged between 0% and 100% with higher scores reflecting a greater preference for acceptance use. Participants with scores of 0% (*n *= 2) and 100% (*n *= 11) were not considered as outliers, as we conceptualized these scores as meaningful individual differences in strategy use preferences.

#### N-back task

Participants completed the *N*-back task (Barch *et al.*, 2013) intra-scanner. The *N*-back consisted of eight blocks of 10 trials in which participants were required to indicate whether the current image matched a previously presented target image. Task blocks alternated between 0-back and 2-back conditions. During 0-back blocks, a single target image was presented at block onset and participants indicated whether each subsequent image matched the target using a two-button response box (match or no match). During 2-back blocks, participants indicated whether the current image matched the image presented two trials prior. Participants completed two runs of the *N*-back task and were shown two of eight possible orders that counterbalanced the block type and image categories (faces, places, tools and body parts).

Accuracy scores for 0-back and 2-back block types were calculated block by block and then averaged across all four blocks, respectively. Average 0-back and 2-back accuracy scores were calculated by computing the average accuracy for each condition (0-back and 2-back) across the two *N*-back runs. Accuracy scores ranged between 0 and 1, such that higher scores reflect greater accuracy. Our analyses focused on 2-back accuracy scores to match how WM performance was operationalized in the derivation study (Avery *et al.*, 2020).

#### Difficulties in Emotion Regulation Scale

Participants completed the Difficulties in Emotion Regulation Scale (DERS; Gratz and Roemer, 2004), which assesses how a person relates to their own emotional experiences. The DERS consists of 36 items in which participants indicate how frequently they experience each statement (e.g. ‘When I’m upset, I have difficulty controlling my behaviors’) using a 5-point Likert scale (1 = ‘almost never’ and 5 = ‘almost always’). Eleven items were reverse-scored before computing a total sum score. Higher total scores suggest greater problems with emotion regulation. Cronbach’s alpha coefficient was 0.91 (95% CI 0.88–0.93), indicating good internal consistency reliability.

#### Mental health status

To establish a baseline profile of mental health status, participants completed the CES-D (Radloff, 1977; Lewinsohn *et al.*, 1997) self-report measure at the in-person screening assessment to assess for depressive symptoms. Participants responded to each of the 20 items using a 4-point Likert scale (0 = rarely or none of the time and 3 = most or almost all of the time), in which higher total sum scores reflect more depressive symptoms. However, participants were deemed ineligible for the study if they received a score $ \ge $20 on the CES-D. Additionally, participants completed the Beck Anxiety Inventory (BAI; Beck *et al.*, 1988) as part of the online self-report questionnaire battery administered at baseline to assess for anxiety symptoms. The BAI consists of 21 items measured using a 4-point Likert scale (0 = not at all, 3 = severely—it bothered me a lot), in which higher total sum scores reflect more symptoms of anxiety.

### Neuroimaging data

Structural and functional MRI data were collected at The Ohio State University Center for Cognitive and Behavioral Brain Imaging using a Siemens Prisma 3-Tesla scanner with a 32-channel head coil. Acquisition parameters can be found in Table 1.

**Table 1.** Image acquisition parameters

Measure	Task	Structural
	*N*-back	T1-weighted
Pulse sequence type	EPI	Gradient echo
Parallel imaging parameters (band type and acceleration factor)	Multiband, 3	Single GRAPPA, 2
Number of volumes	910 (455 × 2 runs)	1
TR	1000 ms	2400 ms
TE	28 ms	2.15 ms
Flip angle	60$^\circ $	8$^\circ $
FOV	240 mm	256 mm
Acquisition matrix	80 × 80 × 45	256 × 256 × 208
Number of slices	45	208
Slice thickness	3.00 mm	1.00 mm
Voxel size	3.00 mm^3^	1.00 mm^3^
Bandwidth (Hz/Px)	2500	220
Echo spacing	0.50 ms	7.2 ms
Acquisition order	Interleaved	Interleaved

*Notes*: TR = repetition time; TE = echo time; FOV = field of view; Hx/Px = hertz/pixel.

#### Preprocessing of functional data

Preprocessing was conducted using fMRIPrep (Esteban *et al.*, 2019): a standardized pipeline that performs minimal preprocessing including brain extraction, motion and distortion correction, field unwarping, normalization and co-registration to corresponding T1-weighted images. Confound files were generated for each participant’s scan and included the following regressors: six rigid body motion parameters, six temporal derivatives and their squares, mean white matter, cerebrospinal fluid, global signal and a single timepoint regressor for outlier timeframes [volumes with framewise displacement (FD) values ≥0.90 mm]. These confounds were regressed from each participant’s preprocessed functional data. Temporal filtering was performed using a high-pass filter of 0.01 Hz to remove slow fluctuating noise (e.g. scanner drift). Finally, spatial smoothing of functional data was performed using a Gaussian-smoothing kernel of 6 mm full width at half maximum.

#### Whole-brain functional connectivity

Whole-brain functional connectivity matrices were constructed for each participant in the current dataset. Parcellation was achieved by first warping a 268-node whole-brain functional atlas (Shen *et al.*, 2013) into each subject’s functional space and then averaging the BOLD time course signal of all voxels within each atlas node for the entire duration of each *N*-back task run. Functional connectivity was then computed as the Pearson correlation between the time course of each node pair (i.e. edge), generating a 268 × 268 matrix of edges for each participant for each *N*-back run. For participants with two usable *N*-back runs, their two correlation matrices were averaged together resulting in a single 268 × 268 matrix; otherwise, only the matrix from the usable *N*-back run was used for participants with excluded runs (see the Statistical analysis section for details). Each participant’s matrix was then normalized using the Fisher *r*-to-*z* transformation.

#### wmCPM networks

To assess the generalizability of the wmCPMs to our dataset, we used the high-WM and low-WM network masks from Avery *et al.* (2020) to calculate a high-WM, low-WM and combined-WM network (high-WM network strength − low-WM network strength) strength score for each participant using custom MATLAB scripts (MATLAB, 2010).

### Statistical analysis

All data were analyzed using RStudio (RStudio Team, 2020). Analyses involving DERS data were not included in the pre-registration and were entirely exploratory. Data collected from 110 older adults was first assessed for incidental findings, missing values, outliers and other errors. *N*-back runs in which the average accuracy was $ \le $0.50 were deemed as outliers and excluded from subsequent analyses (*n*_runs_ = 10). The average 2-back accuracy and overall acceptance use were assessed for normality using the Shapiro–Wilk test (Shapiro and Wilk, 1965). Spearman’s rank-order correlations ($\rho $) were used for non-normally distributed data. Image intensity was dummy-coded (0 = low intensity and 1 = high intensity) to facilitate the interpretation of main and interaction effects. WM network strength and accuracy scores were grand mean-centered to facilitate the interpretation of any potential interaction effects.

In-scanner head motion has been identified as a potential confound in functional connectivity analyses (Power *et al.*, 2012; Van Dijk *et al.*, 2012) particularly among older adults (Haller *et al.*, 2014; Geerligs *et al.*, 2017). However, no participant was excluded for excessive head motion, defined by the mean framewise displacement (FD) values of $ \ge $0.15 mm after preprocessing. Furthermore, we assessed potential relationships between head motion (mean FD) and each behavioral measure (average 2-back accuracy, overall acceptance use, and DERS).

## Results

### Sample demographics

A total of 110 participants completed both the baseline behavioral and neuroimaging assessment sessions (sample demographic descriptive statistics can be found in Table 2). Of these 110 participants, a total of six participants were excluded from all analyses due to incidental findings (*n *= 2) and below-chance performance on both runs of the *N*-back task (*n* = 4). All analyses were conducted with 104 participants, except for analyses using the DERS (*n* = 98) or CES-D (*n *= 98) in which an additional six participants did not complete these self-report measures. We conducted a *post hoc* sensitivity analysis using the WebPower package (Zhang and Yuan, 2018) in R to determine the smallest effect size we would need to observe to detect a significant relationship between WM network strength and acceptance use. Using our final sample size of 104 participants, an alpha of 0.05 and 0.80 power, we would need to observe an effect size (*r*) of 0.27 to detect a significant effect. Similarly, we conducted a *post hoc* sensitivity analysis to determine the smallest effect size we would need to observe to detect a significant network strength × image intensity interaction on acceptance use. For this analysis, using a sample size of 104 participants, three predictors, an alpha level of 0.05 and 0.80 power, we would need to observe an effect size (*f*) of 0.28 to detect a significant effect.

**Table 2.** Descriptive statistics of sample demographics

Variable name	*M* (s.d.) (*N *= 110)	Range (*N *= 110)	*n* (%) (*N *= 110)
**Demographics**			
Age (years)	70.94 (4.64)	65.01–84.17	
Gender (females)			66 (60.00)
Education level (years)	16.94 (2.76)	12.00–27.00	
**Race**			
Asian			1 (0.90)
Black or African American			23 (20.91)
Native Hawaiian or other Pacific Islander			1 (0.90)
White			85 (77.27)
**Ethnicity**			
Hispanic/Latino			1 (0.90)
Non-Hispanic/Latino			109 (99.10)
**Mental health status**			
CES-D	4.25 (4.45)	0–19	
BAI^a^	4.59 (3.93)	0–17	

a BAI questionnaire data were not completed for an additional *n* = 6 participants.

### Behavioral performance and neuroimaging descriptive statistics

**Fig. 1.**
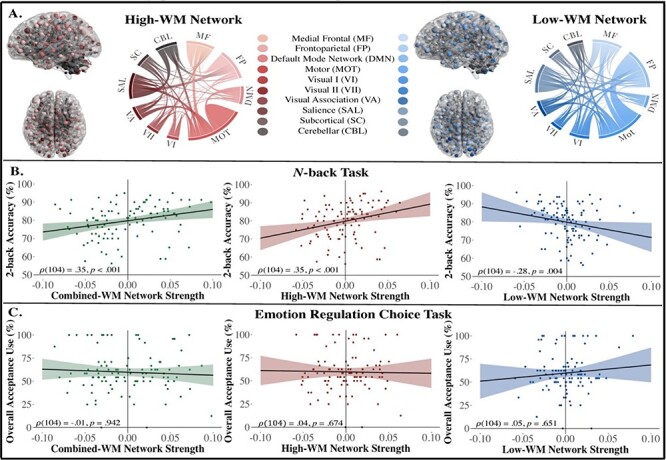
Visualization of the WM networks and their generalizability to WM performance and acceptance strategy use among older adults. (A) Anatomical distribution and contribution of canonical networks to the WM networks. Two nodes in the subcortical and cerebellar networks were excluded, resulting in a total of 1,667 edges in the high-WM network and 1,193 edges in the low-WM network. (B) Spearman’s correlation plots of relationships between WM network strengths and 2-back accuracy on the *N*-back task; accuracy scores displayed in percentages for visualization purposes only; *y*-axis starts at 50 due to exclusion of participants with accuracy scores ≤50%. (C) Spearman’s correlation plots of relationships between WM network strengths and acceptance strategy use. All network strength scores are grand mean-centered and 95% CIs are displayed. Glass brain plots were visualized with BrainNet Viewer (Xia *et al.*, 2013), and chord diagrams were generated using Flourish software.

**Fig. 2.**
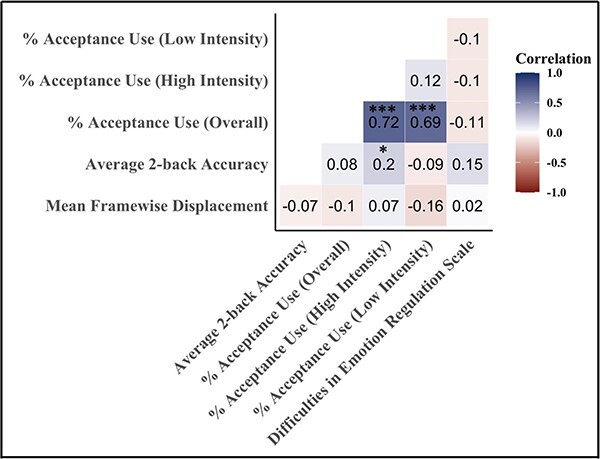
The correlation matrix between head motion and behavioral variables. All reported correlations included *N* = 104 participants unless otherwise noted. *P < 0.05, **P < 0.01 and ***P < 0.001. *n* = 98 for all correlations involving DERS questionnaire data.

Descriptive statistics for the behavioral and neuroimaging variables can be found in Table 3. Four participants were missing two nodes from the subcortical–cerebellar regions, resulting in exclusion of these two nodes for all participants. As such, all neuroimaging analyses were conducted using a 266 × 266 matrix of functional connectivity data (see Figure 1A for network visualization). Correlations between head motion and behavioral variables can be found in Figure 2. Consistent with prior studies using the *N*-back task (Jaeggi *et al.*, 2010; Lamichhane *et al.*, 2020), participants were faster and more accurate on 0-back trials (*M*_RT_ = 1009.36, s.d._RT_ = 200.36; *M*_ACC_ = 0.84, s.d._ACC_ = 0.10) relative to 2-back trials (*M*_RT_ = 1180.33, s.d._RT_ = 201.61; *M*_ACC_ = 0.80, s.d._ACC_ = 0.09). During the ERC task, participants used acceptance more frequently during low-intensity images (*M*_low_ = 64.66, s.d._low_ = 29.00) compared to high-intensity images (*M*_high_ = 54.81, s.d._high_ = 30.74). However, when images were collapsed across intensity types, participants used acceptance ∼60% of the time (*M*_overall_ = 59.74, s.d._overall_ = 21.78). We found no significant associations between mean FD and average 2-back accuracy ($\rho $(104) = −0.07, *P* = 0.478), overall acceptance use ($\rho $(104) = −0.10, *P* = 0.329) or DERS scores ($\rho $(98) = 0.02, *P* = 0.847).

**Table 3.** Descriptive statistics of behavioral and neuroimaging variables

Variable name	*M* (s.d.) (*N *= 104)	Range (*N *= 104)
**N-back task behavioral variables**		
0-back average accuracy	0.84 (0.10)	0.54–0.99
0-back average RT (ms)	1,009.36 (200.96)	715.53–1,670.91
2-back average accuracy	0.80 (0.09)	0.56–0.96
2-back average RT (ms)	1,180.33 (201.61)	688.09–1,786.16
**ERC task behavioral variables**		
Acceptance use: overall (%)	59.74 (21.78)	0.00–100.00
Acceptance use: low intensity (%)	64.66 (29.00)	0.00–100.00
Acceptance use: high intensity (%)	54.81 (30.74)	0.00–100.00
**DERS**		
Total DERS^a^	60.98 (14.46)	36.00–112.00
**Neuroimaging variables**		
Low-WM network strength	0.00 (0.02)	−0.06, 0.08
High-WM network strength	0.09 (0.03)	0.03–0.15
Combined-WM network strength	0.09 (0.04)	0.00–0.19
Mean FD (mm)	0.07 (0.02)	0.04–0.13

*Notes*: The total sample size included 110 participants. However, six participants were excluded due to incidental findings (*n* = 2) and below-chance accuracy on the *N*-back task (*n* = 4), resulting in a final sample of 104 participants.

a DERS questionnaire data were not completed for an additional *n* = 6 participants.

### Generalizability of WM networks on WM performance and emotion regulation measures

#### WM performance

Accuracy scores on the 2-back trials were not normally distributed (*W* = 0.96, *P* = 0.005). As such, all subsequent correlational analyses were conducted using Spearman’s rho (Figure 1B). The network strength score for the combined-WM network was significantly correlated with the average 2-back accuracy ($\rho $(104) = 0.36, *P* < 0.001, 95% CI 0.18–0.51). To determine whether this relationship was driven by network strength scores in the high-WM or low-WM network, we assessed the relationship between each network strength score and average 2-back accuracy separately. The network strength scores in the high-WM network were positively correlated with the average 2-back accuracy ($\rho $(104) = 0.35, *P* < 0.001, 95% CI 0.17–0.51). These results suggest that functional connections predicting better WM performance in the derivation sample (young adults from the Human Connectome Project) were also associated with better WM performance in our independent sample of healthy older adults. Additionally, network strength scores in the low-WM network were negatively correlated with the average 2-back accuracy ($\rho $(104) = −0.28, *P* = 0.004, 95% CI −0.45, −0.09), indicating that the greater functional connectivity that was negatively associated with WM performance in the derivation sample was also associated with poorer WM performance in our sample of older adults.

#### Emotion regulation measures

Overall acceptance use (*W* = 0.93, *P* < 0.001) and DERS scores (*W *= 0.95, *P* = 0.002) were not normally distributed. As such, all subsequent correlational analyses were conducted using Spearman’s rho (Figure 1C). Network strength scores in the combined-WM network were not significantly correlated with overall acceptance use in the ERC task ($\rho $(104) = −0.01, *P* = 0.942, 95% CI −0.20, 0.19). Furthermore, the network strength scores in the high-WM network ($\rho $(104) = 0.04, *P* = 0.674, 95% CI −0.15, 0.23) nor the low-WM network ($\rho $(104) = 0.05, *P* = 0.651, 95% CI −0.15, 0.24) were significantly correlated with overall acceptance use on the ERC task. Similarly, in an exploratory analysis, network strength scores for the combined-WM, high-WM and low-WM networks were not significantly correlated with DERS scores (combined-WM: $\rho $(98) = 0.08, *P* = 0.444, 95% CI −0.12, 0.27; high-WM: $\rho $(98) = −0.04, *P* = 0.716, 95% CI −0.23, 0.16; low-WM: $\rho $(98) = −0.14, *P* = 0.156, 95% CI −0.33, 0.06). To explore if WM, more broadly, was related to either emotion regulation measure in our sample (acceptance use or DERS), we conducted two additional correlations using the average 2-back accuracy instead of WM network strength scores. Our results showed that the average 2-back accuracy was not significantly correlated with acceptance use ($\rho $(104) = 0.08, *P* = 0.417, 95% CI −0.11, 0.27) or DERS scores ($\rho $(98) = 0.15, *P* = 0.153, 95% CI −0.05, 0.33). Taken together, these findings suggest that functional connectivity within edges predictive of WM performance does not successfully generalize to predict acceptance strategy use or emotion regulation difficulties in our sample of healthy older adults. We also failed to find significant relationships between WM behavioral performance and both emotion regulation measures.

### Moderating role of WM networks on the relationship between image intensity and acceptance strategy use

All moderation analyses were conducted with 104 subjects. As shown in Table 4, results from the three linear mixed-effects models showed that combined-WM network strength was not a significant moderator of the relationship between image intensity and acceptance strategy use ($\beta $ = 102.75, *t*(102) = 1.06, *P* = 0.291, 95% CI −86.78, 292.28). However, there was a significant main effect of image intensity such that participants used acceptance less often on high-intensity images ($\beta $ = −9.86, *t*(102) =  −2.46, *P* = 0.016, 95% CI −17.71, −2.00). Similarly, neither the high-WM nor low-WM network strength scores were significant moderators of the relationship between image intensity and acceptance strategy use (high-WM: $\beta $ = 155.10, *t*(102) = 1.07, *P* = 0.286, 95% CI −128.29, 438.48; low-WM: $\beta $ = −108.09, *t*(102) =  −0.62, *P* = 0.538, 95% CI −450.36, 234.19). Although no significant interactions were found, there was again a main effect of image intensity on acceptance strategy use, indicating that older adults tended to use acceptance less on high-intensity images (high-WM: $\beta $ = −9.86, *t*(102) = −2.46, *P* = 0.016, 95% CI −17.71, −2.00; low-WM: $\beta $ = −9.86, *t*(102) = −2.45, *P* = 0.016, 95% CI −17.74, −1.97). At the request of a reviewer, we conducted an exploratory analysis where we replaced WM network strength with the average 2-back accuracy scores and found that the image intensity × average 2-back accuracy interaction term was significant ($\beta $ = 90.03, *t*(102) = 2.14, *P* = 0.034, 95% CI 7.77, 172.29; Table 4). Our *post hoc* simple slope analysis revealed that individuals with lower WM performance (−1 s.d. below the mean) used acceptance less on high-intensity images ($\beta $ = −18.33, *t *= −3.28, *P* < 0.01, 95% CI −29.27, −7.39) compared to individuals with higher WM performance (+1 s.d. above the mean; $\beta $ = −1.38, *t *= −0.25, *P* = 0.80, 95% CI −12.32, 9.56). Together, these findings suggest that WM performance, but not WM network strength, moderated the relationship between image intensity and acceptance strategy use.

**Table 4.** Linear mixed-effects models assessing moderating role of WM networks and behavioral accuracy on the relationship between image intensity and acceptance strategy use

Model	Variable	$\boldsymbol{\beta} $ (SE)	*t*-value	*P*-value	95% CI, LB, UB
Acceptance strategy use ∼ combined-WM + image intensity + combined-WM × image intensity	Intercept	64.66 (2.93)	22.04	<0.001***	58.94, 70.38
	Image intensity	−9.86 (4.01)	−2.46	0.016*	−17.71, −2.00
	Combined-WM	−85.13 (70.80)	−1.20	0.231	−223.20, 52.93
	Combined-WM × image intensity	102.75 (96.75)	1.06	0.291	−86.78, 292.28
Acceptance strategy use ∼ low-WM + image intensity + low-WM× image intensity	Intercept	64.66 (2.94)	22.03	<0.001***	58.94, 70.39
	Image intensity	−9.86 (4.02)	−2.45	0.016*	−17.74, −1.97
	Low-WM	141.71 (127.46)	1.11	0.268	−106.84, 390.25
	Low-WM × image intensity	−108.09 (174.71)	−0.62	0.538	−450.36, 234.19
Acceptance strategy use ∼ high-WM + image intensity + high-WM× image intensity	Intercept	64.66 (2.94)	22.02	<0.001***	58.94, 70.39
	Image intensity	−9.86 (4.01)	−2.46	0.016*	−17.71, −2.00
	High-WM	−92.49 (105.97)	−0.87	0.384	−299.14, 114.16
	High-WM × image intensity	155.10 (144.65)	1.07	0.286	−128.29, 438.48
Acceptance strategy use ∼ average 2-back accuracy + image intensity + average 2-back accuracy × image intensity	Intercept	64.66 (2.91)	22.23	<0.001***	58.99, 70.33
	Image intensity	−9.86 (3.94)	−2.50	0.014*	−17.58, −2.13
	Average 2-back accuracy	−24.81 (30.97)	−0.80	0.424	−85.21, 35.59
	Average 2-back accuracy × image intensity	90.03 (41.99)	2.14	0.034*	7.77, 172.29

*Notes:* Each linear mixed-effects model was conducted using *N* = 104 participants. Image intensity is dummy-coded, and WM network strength and the average 2-back accuracy scores are grand mean-centered. For each model, displayed regression coefficients are unstandardized. LB: lower bound; UB: upper bound.

* *P *< 0.05.

** *P* < 0.01.

*** *P* < 0.001.

## Discussion

The current study examined whether whole-brain networks of WM derived from young adults using CPM generalized to predict WM performance, acceptance use, and emotion regulation difficulties in an independent sample of healthy older adults. We also sought to assess whether WM network strength moderated the relationship between image intensity and acceptance use. Consistent with our hypotheses, functional connectivity strength in the combined-WM network was positively associated with 2-back accuracy in our sample of older adults. *Post hoc* analyses revealed that greater strength in the high-WM network was positively associated with 2-back accuracy, whereas greater strength in the low-WM network was negatively associated with 2-back accuracy. Contrary to our hypotheses, greater strength in the combined-WM network was not significantly associated with acceptance use or emotion regulation difficulties in our sample of older adults. *Post hoc* analyses showed that the non-significant effects on acceptance use and emotion regulation difficulties were replicated for both the high-WM and low-WM networks. Our results also showed that network strength for all three networks did not significantly moderate the relationship between image intensity and acceptance strategy use. To determine whether this null finding was specific to the WM networks or generalized to WM performance more broadly, we conducted an exploratory analysis using 2-back accuracy instead of WM network strength. Here, WM performance (2-back accuracy) moderated the effect of image intensity on acceptance use, such that older adults with lower levels of WM used acceptance less often on high intensity images relative to older adults with higher levels of WM. Consistent with our hypothesis, the wmCPMs successfully predicted individual differences in WM performance in our sample mirroring findings from prior CPM-based studies (Rosenberg *et al.*, 2016; Fountain-Zaragoza *et al.*, 2019; Avery *et al.*, 2020; Manglani *et al.*, 2021; Kardan *et al.*, 2022). Interestingly, some evidence suggests that the performance of predictive modeling approaches is strongest when there is substantial variability in the behavior of interest (Shen *et al.*, 2017), such as in the case of samples containing both healthy adults and individuals with clinical conditions. Therefore, the fact that the wmCPMs successfully generalized to an independent sample of *healthy* older adults demonstrates that the wmCPMs are robust neural markers of individual differences in WM performance.

In contrast to our hypothesis, the wmCPMs did not successfully predict acceptance use during an emotionally evocative behavioral task. Although experimental studies identifying specific cognitive and neural processes underlying acceptance use are relatively non-existent, some theoretical accounts suggest that acceptance falls within the broad category of cognitive change strategies alongside reappraisal-based strategies (McRae and Gross, 2020). Given that well-studied cognitive change strategies (i.e. reappraisal) reliably recruit WM (Hendricks and Buchanan, 2016; Xiu *et al.*, 2018; Powers and LaBar, 2019) and supporting brain networks (Kohn *et al.*, 2014; Morawetz *et al.*, 2017, 2020), we hypothesized that a neuromarker of WM would predict use of acceptance-based strategies. However, some evidence indicates that suppression-based strategies also recruit WM (Hendricks and Buchanan, 2016). As such, it is possible that the recruitment of WM does not significantly differ between acceptance and suppression strategies. To help contextualize this finding, we explored whether this effect was specific to the prioritization of an acceptance *vs* suppression strategy or was extended to a widely used self-report measure of emotion regulation. Similarly, wmCPM network strengths (combined-WM, high-WM and low-WM networks) were not significantly associated with DERS scores, indicating that the wmCPMs were also not predictive of emotion regulation difficulties. Taken together, these findings suggest that the wmCPMs do not successfully generalize beyond cognitive domains to predict emotion-based behaviors.

Prior studies have also found that one’s preferences for and ability to implement cognitive change strategies, namely reappraisal, are moderated by stimulus intensity and WM abilities (Sheppes *et al.*, 2011; Lee *et al.*, 2013; Scheibe *et al.*, 2015). Although wmCPM network strength did not moderate relationships between image intensity and acceptance use, older adults showed a greater preference for acceptance during low-intensity images and suppression during high-intensity images. This finding is comparable to prior studies in which individuals preferred cognitive reappraisal during low-intensity images and distraction during high-intensity images (Sheppes *et al.*, 2011; Scheibe *et al.*, 2015). Interestingly, individual differences in WM performance did moderate the effect of image intensity on acceptance, such that older adults with lower WM used acceptance less often on high-intensity images relative to older adults with higher WM. Given that acceptance of momentary experiences is considered a core mechanism through which mindfulness training reduces affective intensity (Lindsay and Creswell, 2017), our results are similar to our prior work showing that baseline WM abilities moderate the effect of mindfulness training on emotion dysregulation (Samimy *et al.*, 2022). Our constellation of findings in the context of the broader literature suggests that individual differences in WM abilities, but not whole-brain networks of WM, may influence one’s preference for and the effectiveness of using acceptance-based strategies to regulate negative emotions.

### Limitations and future research

Our study was a first attempt at using the pre-existing wmCPMs to predict emotion regulation strategy preferences in older adults, yet our findings were limited by several factors. First, despite some shared features between the strategies employed in prior ERC tasks and those in the current study, the specific cognitive processes and neural correlates that influence preferences for acceptance *vs* suppression strategies remain unclear. Both cognitive reappraisal and acceptance are considered cognitive change strategies that emphasize active engagement with negative emotional states. In contrast, distraction and suppression prioritize disengagement from negative emotional states. As such, strategies that emphasize engagement with unpleasant emotions, particularly during heightened states of arousal, may require greater cognitive resources (e.g. executive control and WM) to successfully down-regulate negative emotions (Lee *et al.*, 2013; Silvers and Guassi Moreira, 2019). Although we found empirical support that WM abilities moderated the relationship between image intensity and strategy use, we did not find evidence that brain-based markers of WM differentially predicted overall acceptance *vs* suppression use. Given that relatively little empirical work exists delineating the specific cognitive and neural processes that are implicated in acceptance-based strategies, it is possible that the neuromarkers of WM used here may not be sensitive enough to detect individual differences in strategy preferences. Future studies should also examine patterns of neural activity during the implementation of acceptance relative to passive viewing or other strategy conditions (e.g. cognitive reappraisal and distraction). Doing so would provide valuable insights into the specific cognitive and neural functions influencing the use of acceptance-based strategies.

Second, social desirability bias and motivations common among older adults potentially confounded clear interpretations of the observed preferences for acceptance during low-intensity images. Older adults tend to behave in socially desirable ways (Dijkstra *et al.*, 2001; Fastame and Penna, 2012); thus, the demand characteristics associated with each strategy may have influenced greater use of acceptance when the subjective cost of doing so was minimal. For example, older adults show a ‘positivity bias’ during emotional processing (see Reed *et al.* (2014) for a meta-analysis) and are typically more motivated to down-regulate negative emotions (Carstensen *et al.*, 2003; Mather and Carstensen, 2005). However, strong negative emotions and heightened states of arousal may be particularly cognitively taxing for older adults (Charles, 2010; Sands and Isaacowitz, 2017). Under such conditions, older adults tend to use less cognitively demanding strategies that help them quickly disengage with strong negative emotions (e.g. distraction and situation selection; Scheibe *et al.*, 2015; Livingstone and Isaacowitz, 2021). Therefore, assuming acceptance is indeed a cognitively costly strategy that encourages engagement with strong negative emotions, its use (although ‘socially desirable’) may be more inconsistent with older adults’ goals and too cognitively taxing for those with lower WM abilities to effectively implement during high-intensity situations. Although this hypothesis is preliminary, the subjective (and objective) cognitive cost of acceptance remains an outstanding question. An interesting and potentially parsimonious way to test whether emotion regulation strategies produce differentiable subjective cognitive costs would be to combine existing ERC tasks (Sheppes *et al.*, 2011) with behavioral economic task paradigms (Westbrook *et al.*, 2013).

Finally, our study used existing wmCPMs to predict strategy use rather than deriving a new neural marker predictive of strategy use. As such, the functional edges associated with WM performance may meaningfully differ from the edges predictive of emotion-based behaviors. It may have been more fruitful to derive CPM networks using functional connectivity data collected during the *N*-back task to predict acceptance use directly. In doing so, both divergent and overlapping edges between the WM performance and acceptance use trained models could be explored. However, work using machine learning models, such as CPM, suggests that large samples (*N* > 100) are needed to derive robust network models (Marek *et al.*, 2022; Shen *et al.*, 2017; but also see Gratton *et al.*, 2022; Rosenberg and Finn, 2022) which the current study was underpowered to do. Although, with the increased availability of large, open-source neuroimaging datasets (Human Connectome Project; Van Essen *et al.*, 2013), future work could be dedicated toward deriving new neural network models predictive of emotion-based behaviors.

## Data Availability

The data used for this article will be made available upon request to the corresponding author.
